# Circularized Nanodiscs for Multivalent Mosaic Display of SARS-CoV-2 Spike Protein Antigens

**DOI:** 10.3390/vaccines11111655

**Published:** 2023-10-28

**Authors:** Moustafa T. Mabrouk, Asmaa A. Zidan, Nihal Aly, Mostafa T. Mohammed, Fadi Ghantous, Michael S. Seaman, Jonathan F. Lovell, Mahmoud L. Nasr

**Affiliations:** 1 Division of Engineering in Medicine and Division of Renal Medicine, Department of Medicine, Brigham and Women’s Hospital, Harvard Medical School, Boston, MA 02115, USA; mmabrouk@buffalo.edu (M.T.M.);; 2 Department of Biomedical Engineering, University at Buffalo, State University of New York, Buffalo, NY 14260, USA; jflovell@buffalo.edu; 3 Schepens Eye Research Institute of Massachusetts Eye and Ear, Department of Ophthalmology, Harvard Medical School, Boston, MA 02114, USA; 4 Botany and Microbiology Department, Faculty of Science, Alexandria University, Alexandria 21526, Egypt; 5 Clinical Pathology Department, Minia University, Minia 61519, Egypt; 6 Center for Virology and Vaccine Research, Beth Israel Deaconess Medical Center, Boston, MA 02115, USA; 7 Broad Institute of MIT and Harvard, Cambridge, MA 02142, USA

**Keywords:** nanodisc, vaccine, COVID-19

## Abstract

The emergence of vaccine-evading SARS-CoV-2 variants urges the need for vaccines that elicit broadly neutralizing antibodies (bnAbs). Here, we assess covalently circularized nanodiscs decorated with recombinant SARS-CoV-2 spike glycoproteins from several variants for eliciting bnAbs with vaccination. Cobalt porphyrin–phospholipid (CoPoP) was incorporated into the nanodisc to allow for anchoring and functional orientation of spike trimers on the nanodisc surface through their His-tag. Monophosphoryl-lipid (MPLA) and QS-21 were incorporated as immunostimulatory adjuvants to enhance vaccine responses. Following optimization of nanodisc assembly, spike proteins were effectively displayed on the surface of the nanodiscs and maintained their conformational capacity for binding with human angiotensin-converting enzyme 2 (hACE2) as verified using electron microscopy and slot blot assay, respectively. Six different formulations were prepared where they contained mono antigens; four from the year 2020 (WT, Beta, Lambda, and Delta) and two from the year 2021 (Omicron BA.1 and BA.2). Additionally, we prepared a mosaic nanodisc displaying the four spike proteins from year 2020. Intramuscular vaccination of CD-1 female mice with the mosaic nanodisc induced antibody responses that not only neutralized matched pseudo-typed viruses, but also neutralized mismatched pseudo-typed viruses corresponding to later variants from year 2021 (Omicron BA.1 and BA.2). Interestingly, sera from mosaic-immunized mice did not effectively inhibit Omicron spike binding to human ACE-2, suggesting that some of the elicited antibodies were directed towards conserved neutralizing epitopes outside the receptor binding domain. Our results show that mosaic nanodisc vaccine displaying spike proteins from 2020 can elicit broadly neutralizing antibodies that can neutralize mismatched viruses from a following year, thus decreasing immune evasion of new emerging variants and enhancing healthcare preparedness.

## 1. Introduction

The COVID-19 pandemic, caused by the novel coronavirus SARS-CoV-2, is a global health crisis that has impacted nearly every aspect of daily life since its emergence in late 2019 [1]. The pandemic has resulted in significant morbidity and mortality, with millions of confirmed cases and deaths reported worldwide [2]. Efforts to combat the pandemic have included vaccine development, social distancing measures, and increased testing and contact tracing. Despite these efforts, the pandemic continues to pose significant challenges to global health and has underscored the importance of international cooperation and public health preparedness [3].

Vaccines are an essential tool in ending the COVID-19 pandemic as they play a crucial role in achieving herd immunity [4]. Although vaccination programs have helped decrease COVID-19 cases, hospitalizations, and deaths, there are still concerns about SARS-CoV-2 viral evolution and immunity evasion mechanisms, which can lead to lower efficacy in the strain-specific vaccines that have been developed [5]. SARS-CoV-2 vaccines show declining efficacy in the face of waning immunity and the emergence of sequence divergent variants such as Omicron, which became the dominant variant worldwide, leading to significant reduction in available vaccine efficacy [6]. Global efforts are underway to develop improved vaccines that can provide broad protection against multiple strains of the SARS-CoV-2 virus and enhance healthcare preparedness. This type of vaccine should ideally address concerns about immune evasion and emerging variants of the virus [7].

Several approaches are being explored to develop a pan-COVID-19 vaccine. One approach is to target a conserved region of the virus, such as the internal nucleocapsid protein, which is less prone to mutation and an immunodominant target [8] and could be used to generate a broad cellular immune responses upon vaccination [9]. On the other hand, studies showed that targeting conserved regions in spike proteins, especially the S2 domain, can result in bnAbs [10,11]. Another approach to produce bnAbs is the use of nanoparticles to display the spike’s receptor binding domain (RBD) antigen from eight different strains of sarbecoviruses on a single particle to enhance immune focusing on the conserved regions of RBD [12,13]. 

The use of advanced materials and nanoparticles can allow association of the antigen with the adjuvants and better presentation of antigens to immune cells [14]. Antigens can be either encapsulated or presented on the nanoparticle surface; encapsulation can provide protection from rapid degradation upon injection while surface conjugation can enhance the immune response by enhancing antigen presentation to immune cells similar to pathogen presentation, thus eliciting a similar response [15]. 

Since its introduction in 2015 [16], Cobalt porphyrin–phospholipid (CoPoP)-based liposomes proved to be an effective vaccine for infectious diseases such as Malaria [17,18,19], Influenza [20], COVID-19 [21,22,23], Lyme disease [24], and enterotoxigenic Escherichia coli [25] as well as cancer [26,27]. Moreover, a CoPoP-based COVID-19 human vaccine has passed phase II clinical trials and is currently tested for phase III [28]. CoPoP is able to present the antigens on the surface of liposomes, thereby converting soluble recombinant proteins into a particle-bound format.

In the present study, a novel circularized large nanodisc was used to anchor various spike proteins through their His-tag via CoPoP lipid. Unlike liposomes, engineered nanodiscs are discoidal in nature and thus all the lipid heads are exposed, allowing more surface for protein display and thus increasing its efficiency as a vaccine delivery agent. Peptide-based and ApoA1-based nanodiscs have been used in vaccine formulations to immunize mice against cancer [29] and influenza [30], respectively. Immune stimulants such as Monophosphoryl lipid A (MPLA) and QS-21 adjuvants were also incorporated into the nanodisc to improve immunogenicity. To combat the potential threat posed by future SARS-CoV-2 variants to global health, we have devised a rationale for the use of mosaic nanodiscs presenting four spike trimers from various distinct SARS-CoV-2 variants. The purpose behind this design is to stimulate the production of antibodies that target epitopes that are conserved among the four spikes. Capitalizing on avidity effects, the B cell receptors (BCR) can recognize the same epitope in different adjacent spikes, enabling BCR cross-linking and subsequent B cell activation. In contrast, nanodiscs featuring homotypic spike trimers are theoretically more likely to engage B cells with BCR that primarily recognize immunodominant accessible spike epitopes, which, though readily identifiable, may be less conserved.

We utilized a mosaic nanodisc displaying spikes from different variants of concern or interest from the year 2020 (WT, Beta, Delta, and Lambda) to immunize mice. Additionally, we evaluated the effectiveness of nanodiscs displaying homotypic antigens (WT, Beta, Delta, Lambda, BA.1, and BA.2 spikes). The resulting sera from the mosaic nanodisc not only efficiently neutralized matching pseudo-typed viruses but also displayed extended efficacy against mismatched pseudo-typed viruses corresponding to variants of concern from the year 2021 (specifically, Omicron BA.1 and BA.2). In contrast, the homotypic formulations only demonstrated neutralization specific to their respective variants.

## 2. Materials and Methods

His-tagged wild-type spike protein was expressed in-house using the human embryonic kidney 293 cells (HEK293) cells. The following reagents were obtained from BEI Resources (Manassas, VA, USA), NIAID, NIH: Spike Glycoprotein (Stabilized) from SARS-Related Coronavirus 2, AY.1 Lineage (Delta Variant) with C-Terminal Histidine and Avi Tags, Recombinant from HEK293 Cells, NR-55710, Spike Glycoprotein (Stabilized) from SARS-Related Coronavirus 2, B.1.1.1 Lineage with C-Terminal Histidine and Avi Tags, Recombinant from HEK293 Cells, NR-55615, Spike Glycoprotein (Stabilized) from SARS-Related Coronavirus 2, B.1.351 Lineage with C-Terminal Histidine and Avi Tags, Recombinant from HEK293 Cells, NR-55310, Spike Glycoprotein (Stabilized) from SARS-Related Coronavirus 2, B.1.1.529 Lineage (Omicron Variant) with C-Terminal Histidine and Avi Tags, Recombinant from HEK293 Cells, NR-56447, Spike Glycoprotein (Stabilized) from SARS-Related Coronavirus 2, BA.2 Lineage (Omicron Variant) with C-Terminal Histidine and Avi Tags, Recombinant from HEK293 Cells, NR-56517. CoPoP were synthesized as previously described [16], Synthetic monophosphoryl Hexa-acyl Lipid A, 3-Deacyl (PHAD-3D6A, Avanti, Alabaster, Al, USA Cat # 699 855), Rhodamine lipid (18:1 Liss Rhod PE, Avanti Cat # 810150C). QS-21 was obtained from Desert King (Chula Vista, CA, USA). FITC was obtained from Sigma (Burlington, MA, USA Cat # F7250). Soluble ACE2-Fc fusion protein was obtained from Invivogen (San Diego, CA, USACat # fc-hace2).

cNW50 expression and purification: Circularized NW50, which is used to generate nanodiscs, was produced as previously described [31]. Briefly, BL21-Gold (DE3) competent *E. coli* cells (Agilent) were transformed using NW50 gene cloned into pET-28a with a tobacco etch virus (TEV) protease-cleavable sequence placed after His tag at the N-terminal. Sortase-recognition sequence was inserted near the C-terminal, thus upon TEV cleavage and sortase circularization, the final circularized NW50 (cNW50) produced is tag-less. Protein production was induced using 1 mM isopropyl β-D-1-thiogalactopyranoside (IPTG) at OD = 0.6. The protein was purified using Ni^2+^-NTA resin. N-terminal His-tag was removed by TEV at 4 °C for 16 h and further purified using size-exclusion chromatography using a Superdex 200 16/60 column. The fractions containing the proteins were pooled and diluted into sortase reaction buffer (150 mM Tris-HCl, pH 7.5, 150 mM NaCl, and 10 mM CaCl_2_). Freshly made evolved sortase (Addgene plasmid # 75144, a gift from David Liu) was added to TEV-cleaved NW50 at molar ratio 1:2 and the reaction was allowed to proceed for 3–4 h at 37 °C under gentle stirring. Circularized NW50 (cNW50) underwent additional purification steps using reverse Ni^2+^ followed by size-exclusion chromatography and ion exchange utilizing a Resource Q column. 

Nanodisc preparation: To assemble cNW50 nanodiscs, various lipid ratios were employed: 1:1000, 1:2000, and 1:4000. These nanodiscs contained a lipid composition of 51% POPC, 34% POPG, 5% Cholesterol, 5% MPLA, and 5% CoPoP. The lipid components, which were solubilized in sodium cholate, were combined with cNW50 and incubated on ice for 1 h. Following the incubation period, the sodium cholate was removed using Bio-beads SM-2 (Bio-Rad, Hercules, CA, USA, Cat # 1523920) overnight at 4 °C. The nanodisc preparations were then filtered through 0.22 µm nitrocellulose-filter tubes to separate the Bio-beads. Subsequently, these preparations were further purified using size-exclusion chromatography while monitoring the absorbance at 280 nm. A Superose 6 10/300 column was employed, and it was equilibrated in PBS. Fractions corresponding to the expected size of each nanodisc were collected and concentrated. The purity of the nanodisc preparations was assessed using SDS-PAGE (Sodium Dodecyl Sulfate–Polyacrylamide Gel Electrophoresis).

Spike-nanodisc preparation: To immobilize the spike protein on the nanodisc, the spike protein was mixed with the nanodisc at (4:1 molar ratio spike: CoPoP) and allowed to shake for 3 h at room temperature. Successful immobilization was confirmed with the shift in the SEC peak on Superose 6 10/300 column and with Ni-beads challenge assay. 

Cell culture: In all experiments, the cells were cultured and maintained in a controlled environment at 37 °C with a humidified atmosphere that contained 5% CO_2_. HEK293T cells were cultured in Dulbecco’s Modified Eagle Medium (DMEM) supplemented with 10% Fetal Bovine Serum (FBS), 1% Penicillin/Streptomycin (Pen/Strep), and 10 × 10^−3^ M sodium pyruvate. THP-1 cells, on the other hand, were cultured in antibiotic-free RPMI-1640 media. This medium was further supplemented with 0.05 mM 2-mercaptoethanol, 10% FBS, and 1% Penicillin/Streptomycin (Pen/Strep).

In vitro macrophage uptake and polarization assay: THP-1 cells were used as a model for macrophages to test uptake and macrophage polarization. THP-1 cells were seeded at a concentration of 3 × 10^5^ cells/mL and maintained at 37 °C, 5% CO_2_ in a humidified tissue culture incubator. THP-1 cells were primed to macrophage-like cells through treatment with 100 ng/mL PMA for a 24 h priming period, followed by washing PMA off and resting for 72 h. To test the uptake of the nanodisc (ND) and spike proteins, cells were incubated with 2 µg/mL fluorescently labeled spike protein, fluorescently labeled ND, or double-labeled spike–ND complex for 18 h (*n* = 3). Cells were then collected and analyzed by flow cytometry. To test the effect of ND on macrophage polarization into M1, cells were then incubated with spike/ND or spike alone for the 48 h polarization period. Finally, the cells were stained using anti-CD86 APC as M1 markers and analyzed by flow cytometry (*n* = 3). TNF-α production was quantified using commercial ELISA kit (ThermoFisher, Waltham, MA, USA Cat # 88-7346-22) according to manufacturer guidelines. All samples were measured 3 times and data were analyzed with one-way ANOVA.

Ni-NTA bead competition assay: To assess the stability of spike protein binding in its particle form, we followed the procedures as previously outlined in [22]. In this process, Ni-NTA magnetic beads (ThermoFisher Cat # 88831) were employed to compete with CoPoP on spike protein, which was pre-bound to the nanodisc. In brief, a sufficient amount of Ni-NTA magnetic beads was added to ensure complete binding of all spike proteins in the sample. The samples were then incubated with these beads for 30 min at room temperature (RT). Subsequently, the nanodisc–spike complex and the magnetic beads (carrying unbound spikes) were separated and collected using a magnetic separator (ThermoFisher Cat # 12321D). The collected beads were resuspended in PBS. Denaturing reducing loading dye was added to all the samples (both supernatant and beads), and the samples were heated at 95 °C for 10 min. They were then loaded onto 4–20% Mini-PROTEAN^®^ TGX™ Precast Protein Gels, 15-well, 15 µL (Biorad, Cat # 4561096). Subsequently, the samples underwent polyacrylamide gel electrophoresis (PAGE), and the protein bands were visualized through Coomassie staining [22].

Slot blot assay: In this procedure, 50 µL of spike protein, either in its free form or bound to nanodiscs, was applied to a 96-well dot blot apparatus, following the manufacturer’s instructions. The samples were gently introduced into each well and allowed to pass through a 0.45 µm nitrocellulose membrane (GE Healthcare, Chicago, IL, USA Cat # 10600096) under vacuum. Afterward, the membrane was carefully removed and subjected to blocking with a 5% BSA solution in PBS for 30 min at room temperature (RT). Subsequently, the membrane was incubated with a 1000-fold diluted solution of human ACE2-Fc Tag (Cat # AC2-H5257 from ACRO Biosystems, Newark, DE, USA) for 1 h at RT. The membrane was washed with PBS for 5 min, repeated twice. Following the washing step, the membrane was incubated with HRP anti-human IgG (Cat # 109-035-098 from Jackson ImmunoResearch, West Grove, PA, USA) for 30 min at RT. After this incubation, the membrane was washed with PBS for 5 min, twice. The membrane was then developed using an HRP substrate (VisiGlo™ HRP Chemiluminescent Cat # 97064-146) and subsequently imaged using a Bio-Rad ChemiDoc Imager (Hercules, CA, USA).

Murine immunization: All procedures related to animal care and sample collection were conducted with the approval and in accordance with the guidelines of the Institutional Animal Care and Use Committee at Brigham and Women’s Hospital. To assess the immunogenicity of mosaic nanodiscs incorporating four spike variants, a comparison was made with nanodiscs containing a single spike variant or empty nanodiscs. This assessment was carried out in CD-1 mice, following previously established procedures [22]. Mice were divided into 8 groups (*n* = 5); 6 homotypic nanodisc carrying single antigens (WT, Beta, Delta, Lambda, BA.1, and BA.2), a mosaic nanodisc (WT, Beta, Delta, Lambda), and a control group with no antigen (nanodisc without spikes). On days 0 and 14, the mice were administered intramuscular injections. These injections included 0.2 µg of total spike protein bound to nanodisc. The nanodisc vaccines were prepared by incubating the spike protein at a concentration of 1 mg mL^−1^ with nanodisc (equivalent to a CoPoP concentration of 250 µg mL^−1^) for 3 h at room temperature (RT). Subsequently, the vaccine was diluted to a final concentration of 4 µg/mL with sterile PBS, and each animal received a 50 µL intramuscular injection. Serum samples were collected on day 28 for further analysis.

ELISA: To coat the plates, 1 µg mL^−1^ of spike protein from various variants was diluted in a coating buffer (comprising 28.5 mM Na_2_CO_3_ and 71.4 mM NaHCO_3_, pH 9.6). These solutions were used to coat 96-well plates, which were incubated for 2 h at 37 °C. Subsequently, the wells were washed and blocked with a solution containing 2% BSA in PBS supplemented with 0.1% Tween-20 (PBS-T) for 2 h at 37 °C. For the serological analysis, mouse sera from 8 immunized groups were diluted in PBS-T (1:200) and then was 10 folds serially diluted, which also contained 1% BSA. These dilutions were then added to the wells and allowed to incubate for 1 h at 37 °C. Following this incubation, the wells were washed with PBS-T. To detect the antibodies, goat anti-mouse IgG-HRP (Catalog # A00160 from Genscript, Piscataway, NJ, USA) was introduced into the wells. Afterward, the wells were washed again with PBS-T before the addition of a tetramethylbenzidine (TMB) solution (Catalog # J60461 from Thermo Fisher Scientific). Antibody titers were determined as the reciprocal serum dilution at which the absorbance at 450 nm exceeded the background level by more than 0.5 absorbance units. Results were analyzed using a one-way ANOVA test, followed by Tukey’s comparisons. 

Spike/RBD-hACE2 Inhibition Assay: In this experiment, SARS-CoV-2 spike proteins were utilized to coat 96-well plates at a concentration of 0.5 µg/mL. This coating process was carried out at 4 °C overnight. Subsequently, the plates were blocked with a 2% BSA solution for 2 h at 37 °C. The objective was to assess whether post-immune sera had the ability to inhibit the interaction between hACE2-Fc and the spike protein coated on the plate. The sera were diluted 20-fold with PBST (PBS with Tween-20). For the positive control, PBST with naïve sera was added, while for the negative control, unrelated gp160 (an HIV trimer) was used to coat the wells. The diluted samples were mixed with a solution of Fc-hACE2 (at a concentration of 0.5 µg/mL) at a 1:1 volume ratio and incubated at 37 °C for 30 min. Following this, 100 µL of these mixtures was added to an ELISA plate that had been pre-coated with spike proteins from different variants. The plate was incubated at 37 °C for 15 min. Subsequently, the plate was washed four times to remove any unbound Fc-hACE2. HRP-antiFc was added and incubated at 37 °C for 30 min, followed by six washes. Afterward, a TMB solution was added, and the absorbance was measured at 450 nm using a microplate reader. The percentage of inhibition was calculated using the following formula: % = (1 − OD450 post-immune sera/OD450 negative control) × 100. The assay is explained with a schematic in Figure S3. Samples were tested in triplicates and results were analyzed using one-way ANOVA test, followed by Tukey’s comparisons. 

Pseudo-typed SARS-CoV2 Virus (PsV) neutralization assay: The measurement of neutralizing activity against the SARS-CoV-2 pseudovirus was conducted using a single-round infection assay in 293T/ACE2 target cells. Pseudotyped virus particles were generated in 293T/17 cells (ATCC) through the co-transfection of plasmids encoding codon-optimized full-length spike, the packaging plasmid pCMVR8.2, and the luciferase reporter plasmid pHR’ CMV-Luc. The packaging and luciferase plasmids were generously provided by Dr. Barney Graham from the NIH Vaccine Research Center. The 293T cell line, which stably overexpresses the human ACE2 cell surface receptor protein, was provided by Drs. Michael Farzan and Huihui Ma from The Scripps Research Institute. For the neutralization assays, mouse sera were serially diluted in duplicate, followed by the addition of the pseudovirus. As positive and negative controls, pooled serum samples from convalescent COVID-19 patients and pre-pandemic normal healthy serum (NHS) were used, respectively. The assay plates were incubated for 1 h at 37 °C, after which 293/ACE2 target cells (1 × 10^4^/well) were added. Wells containing cells with pseudovirus (without a sample) and wells with cells alone served as positive and negative infection controls, respectively. The assays were harvested on day 3 using Promega BrightGlo luciferase reagent, and luminescence was detected with a Promega GloMax luminometer. Titers are reported as the serum dilution that inhibited 50% or 80% of virus infection, known as ID50 and ID80 titers, respectively. Results were analyzed using a one-way ANOVA test, followed by Tukey’s comparisons.

Electron microscopy: Nanodisc–spike samples were applied to carbon-coated copper grids that had been glow-discharged and subsequently stained using a 0.75% (*w*/*v*) uranyl formate solution. Electron microscopy (EM) images were captured using a Philips CM10 electron microscope, which was equipped with a tungsten filament and operated at an accelerated voltage of 100 kV. The images were recorded using a Gatan 1K × 1K CCD camera (Pleasanton, CA, USA)

Fluorescent quenching assay: The FITC-labeled spike/nanodisc was prepared by co-incubating antigens and Nanodisc in a 4:1 molar ratio. This incubation was carried out at room temperature (RT) for 3 h, with the final CoPoP concentration set at 250 µg mL^−1^. The quenching of each sample was assessed at multiple time points, specifically at 0, 0.25, 0.5, 1, 2, and 3 h. To evaluate the fluorescence signal, each of the incubated samples was diluted 1:200 in PBS and placed in a 96-well plate. The fluorescence signal was measured at an excitation/emission wavelength of 495/519 nm using a microplate reader. The percentage of antigen binding was calculated using the following formula: % antigen binding = [1 − *FL*_Nanodisc+antigen_/*FL*_antigen_] × 100% where *FL* = fluorescent intensity [32]. Each sample was tested 3 times.

Serum Stability: The FITC-labeled spike and CoPoP nanodisc mixture was incubated for a period of 3 h at room temperature (RT). Following this incubation, an equivalent amount of 40% human serum in PBS was added to the sample, resulting in a final serum concentration of 20%. The samples were subsequently incubated at 37 °C for the specified durations and tested 3 times.

Fluorophore-Labeled Spike: The process of labeling spike with FITC was conducted at RT and involved a molar ratio of 10:1 (FITC to spike). To begin, 100 µg of spike was dialyzed against a 100 × 10^−3^ M sodium bicarbonate buffer with a pH of 9. This dialysis was carried out for 4–6 h at 4 °C, repeated twice. Subsequently, the dialyzed spike was labeled with FITC for 1 h at RT with continuous stirring. To eliminate any unbound dye, a spin column with a 40,000 MW limit was employed, and the buffer was replaced with PBS.

## 3. Results and Discussion

**Figure 1:**
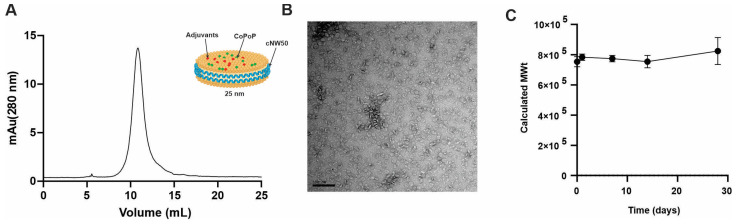
Formation of stable nanodisc using cNW50 incorporating CoPoP as a His-tag protein-binding lipid, and QS-21 and MPLA as immune stimulants. (**A**) Size exclusion chromatogram of empty nanodiscs containing MPLA, QS-21, and CoPoP, with a schematic insert of the proposed structure of the formed nanodisc. (**B**) Negatively stained transmission electron micrograph of formed nanodiscs, scale bar = 100 nm. (**C**) Stability of formed nanodisc at 4 °C for 28 days as tested by size exclusion chromatography.

We previously reported that His-tagged spike or its RBD protein can bind stably to CoPoP-containing liposomes upon liquid admixing, which rendered it an effective immunogen in mice [21,22]. In this study, CoPoP was incorporated into a 25 nm covalently circularized nanodisc to examine whether a mosaic nanodisc decorated with spike proteins from different variants can direct the immune system to produce broadly neutralizing antibodies thanks to dilution of immune dominant, variant specific epitopes with little to no effect on the conserved epitopes and thus favoring immune-recessive conserved epitopes [33]. The plug-and-display approach enabled by CoPoP technology allowed a straightforward production of mosaic nanodisc with different spikes attached randomly [26,34]. We previously reported that 1:4000 cNW50: lipid ratio was used to form a 50 nm nanodisc when 3:2 POPC: POPG lipids were used [31]. After changing the lipid content to accommodate (5% CoPoP, 5% MPLA and 5% Cholesterol), the 1:4000 ratio resulted mainly in aggregation/liposomal formation rather than nanodisc as confirmed by SEC (Figure S1). By lowering the ratio to 1:2000, a 25 nm nanodisc was formed as the major component, and further reduction of the ratio to 1:1000 resulted in a 25 nm nanodisc together with some non-lipid associated cNW50. Upon purifying the peak corresponding to 25 nm nanodisc and re-running SEC, a single peak chromatogram was obtained (Figure 1A). The collected peak was examined using negative staining electron microscopy, which revealed homogenous 25 nm nanodisc (Figure 1B). The assembled nanodisc showed good shelf stability for at least 28 days when stored at 4 °C with no apparent aggregates (Figure 1C).

**Figure 2:**
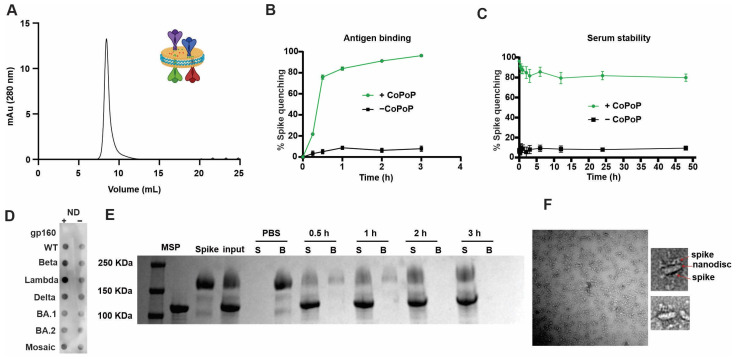
Recombinant, His-tagged spike proteins bind to CoPoP nanodiscs. (**A**) Size exclusion chromatogram of spike bound nanodisc using a Superose 6 column, with schematic insert of the proposed structure of the formed nanodisc. (**B**) Binding kinetics of a fluorescent labeled spike protein to CoPoP nanodisc. (**C**) Binding stability of a fluorescent labeled spike decorated nanodisc when incubated with 20% serum for 48 h in the dark at 4 degrees. (**D**) Dot blot detection of ACE2 binding to adsorbed spike or gp160 (an unrelated control antigen) in soluble or particulate form. (**E**) Nickel- nitrilotriacetic acid (Ni-NTA) bead competition assay; spike protein antigen was incubated with CoPoP nanodisc for various times, and then Ni-NTA beads were added and then isolated for analysis. Protein that was stably bound to nanodisc is in the supernatant (“S”) lanes, whereas the unbound protein is in the bead (“B”) fraction. (**F**) TEM image of spike decorated nanodisc, scale bar = 100 nm.

Recombinant spike antigens from various SARS-CoV-2 variants were used to decorate the nanodisc containing CoPoP, PHAD-3D6A, and QS-21 (Figure 2A). PHAD-3D6A is an MPLA, which is well established as a TLR4 activator and has the capacity to trigger a robust immune response characterized by type-1 CD4 T-helper cells (Th1) [35]. This Th1 response is of utmost importance in facilitating the development of high-affinity antibodies. QS-21 stimulates Th2 humoral and Th1 cell-mediated immune responses in vivo by affecting antigen presenting cells (APCs) and T cells. This leads to the release of Th1 cytokines, which play a role in combating intracellular pathogens such as viruses [36]. When combined, MPLA and QS-21 create a distinctive synergy that elevates the initial IFN-γ response and enhances the immunogenicity of the vaccine [37]. MPLA and QS-21 are powerful adjuvants, which have been proven safe and effective in clinical trials. They have been used in liposomal adjuvants such as AS01 by GSK as a malaria vaccine [38]. Upon binding of the spike protein to the nanodisc for 3 h at room temperature, the formulation was subjected to sizing SEC where the peak showed a significant shift to larger size, eluting at 8.5 mL instead of 11 mL reflecting an increase in apparent size (Figure 2A). The resultant spike-decorated nanodisc was examined by SDS-PAGE showing both bands for spike trimer and cNW50. EM revealed 25 nm nanodiscs without large aggregates, concluding that the spike protein was efficiently bound to the nanodisc at a molar ratio of 4:1 (Spike: Nanodisc) (Figure 2F).

To test the stability of the bound spike protein to the nanodisc, a fluorescence resonance energy transfer (FRET) assay was developed using a fluorescent-labeled spike, which is quenched upon binding to the nanodisc due to energy transfer to the CoPoP chromophore [39]. As shown in (Figure 2B,C), following the binding of the fluorescently labeled spike protein to the nanodisc, the labeled spike formed serum-stable antigen-nanodisc particles. Fluorescence quenching indicated that the antigen was still maintained in the form of intact particles after 48 h incubation with 20% human serum at 37 °C.

Furthermore, to test binding stability of antigen to the nanodisc, the nanodisc was challenged by addition of Ni-NTA magnetic beads, as nickel has a higher affinity to his-tagged protein than cobalt. Figure 2E shows the kinetics of binding of the spike protein to the nanodisc, where Ni-NTA beads were not able to extract the protein from the nanodisc. This can reflect its stability upon injection allowing codelivery of antigen and adjuvants to antigen-presenting cells, which can be crucial for the quality of the produced antibodies [40].

The conformational integrity of the spike in the nanodisc can be of high importance to the quality of the produced antibodies, especially against conformational epitopes. CoPoP lipid allowed simple and stable presentation or spike antigens on the surface of the nanodisc with simple mixing without the need for chemical conjugation or a harsh environment. Moreover, CoPoP can ensure proper antigen orientation as it binds to the His-tag present only at the tail of the spike, mimicking its orientation on the viral surface. To assess confirmational stability and proper orientation, a dot blot assay was developed where either the free spike or nanodisc–spike were adsorbed to a nitrocellulose membrane and then was probed with hACE2-Fc. An HRP-secondary anti-human fc antibody was then used to detect ACE2. ACE2 recognized the spike in both particulate (bound to nanodisc) and free soluble form (Figure 2D), showing that the spike maintains the right conformation and ability to bind to ACE2 after binding to the nanodisc. Human immune deficiency virus Envelop trimer (gp160) was used as an irrelevant control viral trimer and showed no binding to ACE-2.

**Figure 3:**
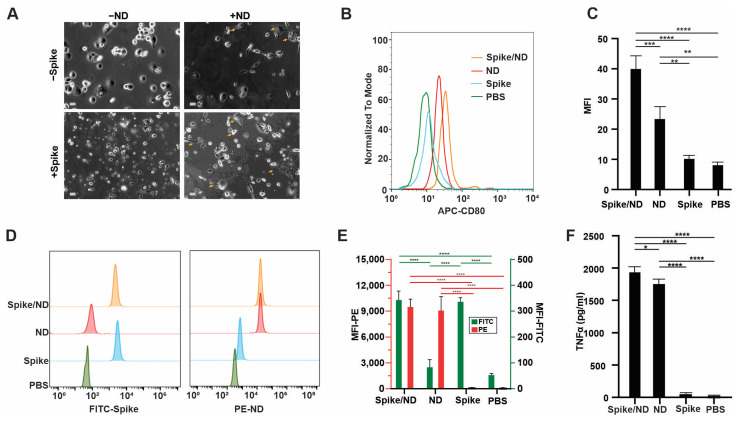
Spike/CoPoP nanodisc is uptaken by THP-1 macrophage cells and favors M1 differentiation. (**A**) Microscopy photos showing polarization of PMA differentiated THP-1 from M0 to M1 cells in response to ND and/or spike addition, yellow arrows show M1-polarized macrophages. (**B**) Flowcytometry histogram of CD 80 indicating polarization of M0 macrophages to M1 due to MPLA and QS-21 incorporated into nanodisc. (**C**) Analysis of mean fluorescence intensity of M1-polarized cells. (**D**) Flowcytometry histogram of uptake of PE-labeled nanodisc and/or FITC-labeled spike protein. (**E**) Analysis of mean fluorescence intensity of uptake of PE-labeled nanodiscs (left *y*-axis, red bars and stats) and/or FITC-labeled spike protein (right *y*-axis, green bars and stats). (**F**) TNF-α release from macrophages as measured by ELISA in response to addition of nanodisc alone, spike alone and nanodisc-spike. Analysis was performed by one-way ANOVA test, followed by Tukey’s comparisons, * *p* < 0.05, ** *p* < 0.01, *** *p* < 0.005 and **** *p* < 0.001.

Phagocytosis plays a crucial role in the immune system, as it involves the uptake and processing of antigens by macrophages and dendritic cells, especially when it comes to particulate vaccine delivery systems’ ability to co-localize antigen and adjuvant [41]. To investigate the uptake of the free spike protein or nanodisc-spike complexes by immune cells, a 0.5% rhodamine-labeled lipid was added to the nanodisc preparations to be able to track its uptake. In vitro studies were performed with THP-1 after differentiation to resting macrophages (M0) by incubation with 100 ng/mL PMA. M0 cells were polarized into M1 cells upon incubation with MPLA and QS-21-containing nanodisc, which can be observed as cells having dendritic-like with filopodia or cytoplasmic extrusions (Figure 3A). Cellular uptake was assessed with flowcytometry upon incubation of cells with an FITC-labeled spike, rhodamine-labeled nanodisc, or FITC-labeled spike decorated rhodamine nanodisc. (Figure 3D,E). As spike trimer is a relatively large protein, it was well uptaken by the cells and we did not observe enhancement in the uptake when the spike was admixed with the nanodisc. The uptake of the spike can can be also attributed to the fact that M1 macrophages upregulate ACE-2 receptor and thus facilitate the uptake [42]. Creating a pro inflammatory microenvironment at the injection site and draining lymph node is desirable and can potentiate the vaccine effect [43], thus, we investigated the ability of the vaccine preparations but not spike alone to polarize resting macrophages into proinflammatory M1 macrophages. When M0 macrophages were incubated with MPLA and QS-21-containing nanodiscs, expression of an M1-specific marker CD80 was upregulated (Figure 3B,C). Moreover, spike itself can augment the action of TLR4 agonists, such MPLA, enhancing the activation of macrophages and TNF-α production through its interaction with ACE-2 receptor [44]. On the other hand, when M0 macrophages were incubated with Spike alone, no significant activation occurred. Thus, the combination and colocalization of the spike protein and nanodisc resulted in enhancement of activation and production of TNF-α (Figure 3F). 

**Figure 4:**
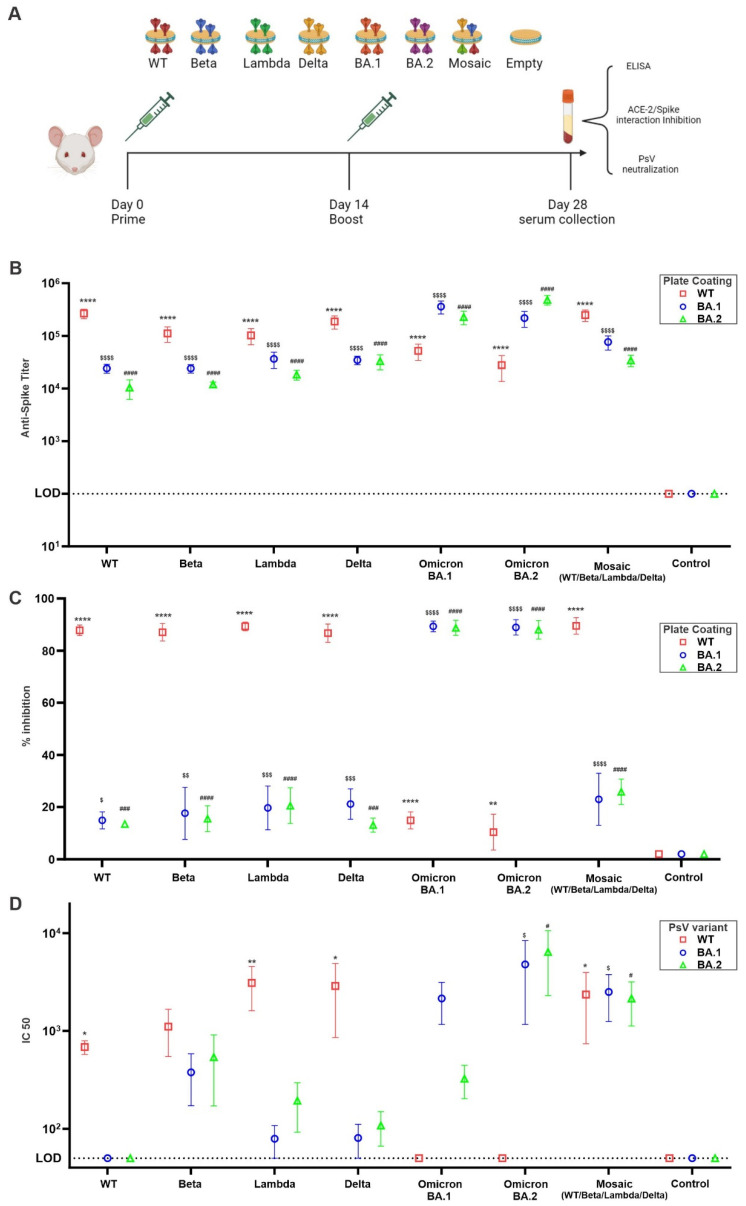
Broadly neutralizing antibodies induced by spike nanodiscs. (**A**) Schematic representation of immunization scheme and immunogens used. CD-1 mice (*n* = 5 per group) were immunized as indicated. (**B**) Anti-spike ELISA IgG titer using WT, BA.1, or BA.2 as coating antigen (**C**) WT, BA.1, or BA.2 spike/ACE-2 interaction inhibition by immunized mice sera after 1:20 dilution. (**D**) Pseudo-typed virus neutralization assay using WT, BA.1, or BA.2 decorated pseudo-typed virus. For (**B**,**D**), log10-transformed titer was analyzed by one-way ANOVA test, followed by Tukey’s comparisons. For (**C**), data were analyzed by one-way ANOVA test, followed by Tukey’s comparisons. * *p* < 0.05, ** *p* < 0.01 and **** *p* < 0.001 against WT control; ^$^ *p* < 0.05, ^$$^ *p* < 0.01, ^$$$^ *p* < 0.005, and ^$$$$^ *p* < 0.001 against BA.1 Control; ^#^ *p* < 0.05, ^###^ *p* < 0.005, and ^####^ *p* < 0.001 against BA.2 Control.

To assess the immunogenicity and the ability of the nanodisc preparations to elicit non-RBD binding bnAbs, we immunized outbred CD-1 mice (*n* = 5) intramuscularly in a prime-boost regimen with 0.2 µg total antigen dose (Figure 4A). Seven immunogens were used in the immunization (WT, Beta, Lambda, Delta, BA.1, BA.2, and mosaic). The first six immunogens are single spike bound to the nanodisc while the seventh (mosaic) is a mixture of equal amounts of spikes (WT, Beta, Lambda, and Delta) bound to nanodisc. Empty nanodiscs were used as a control. All groups elicited high spike-specific IgG antibody titers when tested by ELISA coated by the same antigen of immunization (Figure 4B). 

In our effort to address the potential threat of future SARS-CoV-2 variants to global health, we have prepared and tested nanodiscs designed to showcase spike trimers from four distinct SARS-CoV-2 variants (mosiac). The aim is to stimulate the production of antibodies targeting epitopes that are conserved and somewhat concealed, rather than those that are variable, highly immunogenic, and readily exposed. The likelihood of adjacent spikes being identical is deliberately kept low in the mosaic nanodisc design. This feature is chosen to promote interactions and activation of B cells equipped with BCR that can recognize the same epitope in neighboring, different antigens, allowing cross-linking between BCR and subsequently B cell activation. In contrast, nanodiscs presenting homotypic Spike trimers are theoretically more inclined to engage B cells with receptors that predominantly recognize immunodominant and sterically accessible, yet less conserved, Spike epitopes. To test if the mosaic nanodisc was able to shift the immune focusing from immune dominant mutating RBD to conserved non-RBD epitopes, we assessed the ability of post-immunized sera to inhibit RBD/hACE-2 interaction. As shown in (Figure 4C), sera from homotypic antigen immunized mice were able to inhibit more than 80% of the RBD/hACE-2 interaction in crossmatched antigens but decreased to less than 40% against mismatched antigens. Sera from mosaic nanodisc vaccinated mice did not show enhanced inhibition in RBD/hACE-2 interaction when tested against mismatched antigens (Omicron BA.1 and BA.2). On the other hand, only sera from mosaic nanodisc vaccinated mice were able to neutralize mismatched pseudo-typed viruses (Figure 4D). Homotypic immunized mice sera were only capable of neutralizing cross-matched pseudo-typed viruses. Taken together, our data suggests that BA.1 and BA.2 pseudo-typed virus neutralization by mosiac nanodisc was not due to RBD inhibition and probably was due to Ab binding to extra-RBD neutralizing epitopes. This effect might be attributed to the ability of the mosaic nanodisc to only crosslink B-cells able to bind to multiple antigens on mosaic nanoparticles [12,13] and also the presentation of several variants of the same antigen can subvert immunodominance of mutating epitopes by local dilution in APCs endosomes following phagocytosis of the nanodisc. Interestingly, sera from mice immunized with beta variant spike trimer showed some elevated, yet non-significant, activity against omicron pseudo viruses (Figure 4D). Epitope mapping can be used to examine and confirm the increased targeting of the mosaic and, to a lesser extent, the Beta variant vaccination to the conserved, non-RBD epitopes.

## 4. Conclusions

We formulated a mosaic nanodisc from early 2020 SARS-CoV-2 variants that can show neutralizing activity against latter variants from 2021 (Omicron). Mosaic nanodisc-induced antibodies appeared to have activity beyond inhibition of the interaction of the virus with its receptor and possibly by inhibiting more the conserved fusion machinery on the spike. To our knowledge, this is the first study describing the use of highly stable large circularized nanodisc as a vaccine adjuvant system benefiting from its ability to accommodate several copies of large proteins such as the spike trimer. The strategy described in this study has the potential to mitigate the influence of immune-evading variants on existing vaccines. Furthermore, it may aid in the creation of more resilient vaccines, thereby reducing the strain on healthcare systems and decreasing hospitalizations when confronted with immune-evading variants, like the Omicron variant. Our study also proposes that incorporating a broader range of variants into the nanodisc structure could bolster its effectiveness against emerging variants. It is worth noting that there are some limitations to this study. The membrane stabilizing scaffold being used to assemble the nanodisc is derived from human ApoA1, thus our immunogens can result in eliciting self-antibodies and can cause autoimmunity. Further engineering of cNW50 to diminish its immunogenicity while maintaining its ability to assemble nanodiscs, can lead to nanodiscs with reduced risk of autoimmune disease. Another limitation of our study is the mosaic vaccine was not tested in an animal viral challenge model, which would confirm its efficacy in vivo.

## Data Availability

All data needed to evaluate the conclusions in the paper are present in the paper and/or the Supplementary Materials.

## Supplementary Materials

The following supporting information can be downloaded at: https://www.mdpi.com/article/10.3390/vaccines11111655/s1. Figure S1: Effect of lipid: MSP molar ratio on formation of 25 nm nanodisc, Figure S2: Anti MSP IgG titer measured by ELISA, Figure S3: A schematic figure showing spike/RBD-hACE2 Inhibition Assay.
