# Supplementary material for: Circularized Nanodiscs for Multivalent Mosaic Display of SARS-CoV-2 Spike Protein Antigens
Source: Vaccines (Basel). 2023 Oct 28;11(11):1655. doi: 10.3390/vaccines11111655 (PMC10675430; doi:10.3390/vaccines11111655)
Supplement: Supplementary file 1 [file vaccines-11-01655-s001.zip › vaccines-2665814-supplementary.pdf]

Circularized Nanodiscs for Multivalent Mosaic Display of SARS-CoV-2 Spike Protein Antigens

Moustafa T. Mabrouk [1,2], Asmaa A. Zidan [3], Nihal Aly [1], Mostafa T. Mohammed [1,4], Fadi Ghantous [5], Michael S. Seaman [5], Jonathan F. Lovell [2] and Mahmoud L. Nasr [1,6,*]

Affiliations:

Division of Engineering in Medicine and Division of Renal Medicine, Department of Medicine, Brigham and Women's Hospital, Harvard Medical School, Boston, MA 02115, USA; mmabrouk@buffalo.edu (M.T.M.)

[2] Department of Biomedical Engineering, University at Buffalo, State University of New York, Buffalo, NY 14260, USA; jflovell@buffalo.edu

[3] Schepens Eye Research Institute of Massachusetts Eye and Ear, Department of Ophthalmology, Harvard Medical School, Boston, MA 02114 USA

[4] Clinical Pathology Department, Minia University, Minia, Egypt

[5] Center for Virology and Vaccine Research, Beth Israel Deaconess Medical Center, Boston, MA 02115, USA

[6] Broad Institute of MIT and Harvard, Cambridge, MA 02142, USA

* Correspondence: mnasr@bwh.harvard.edu

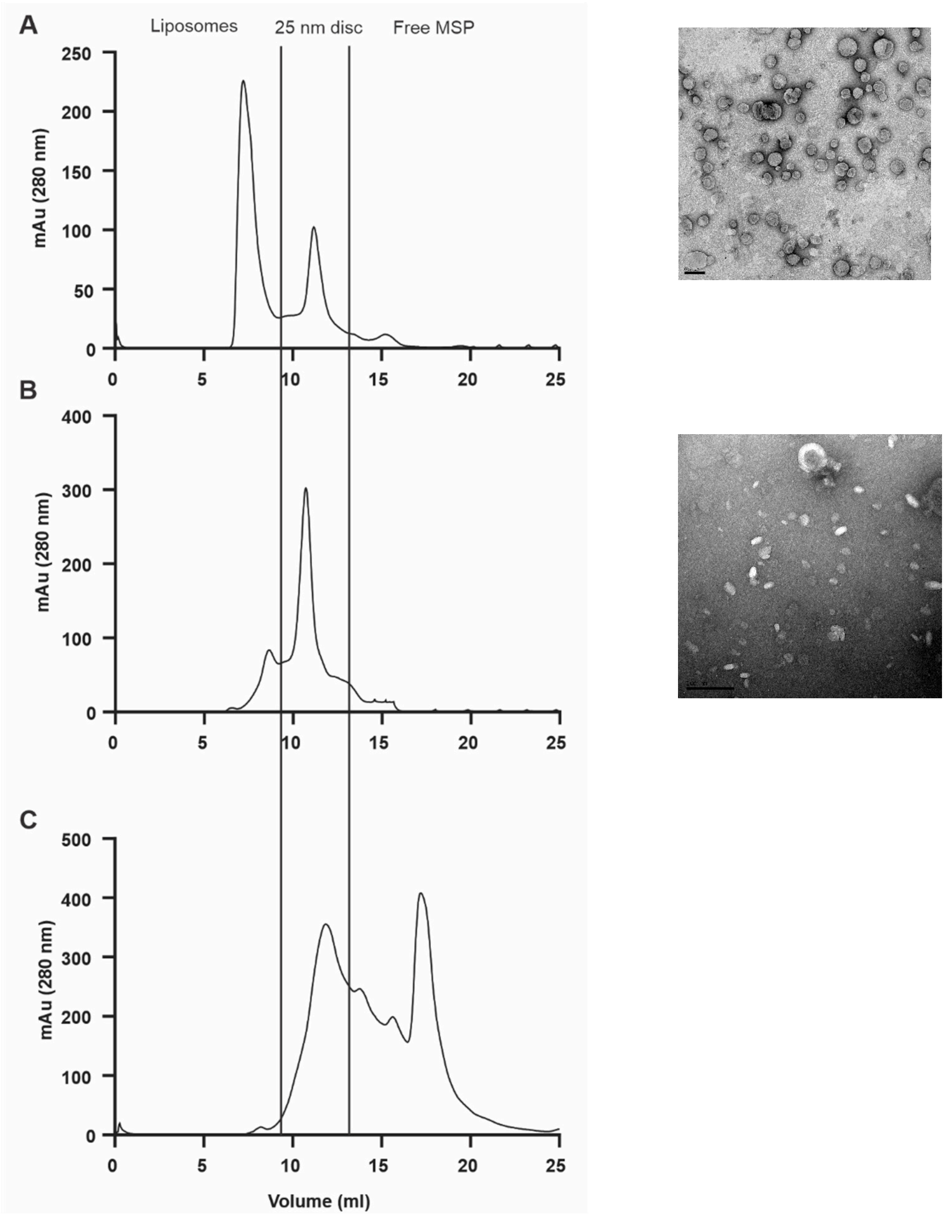

Figure S1. Effect of lipid: cNW50 molar ratio on formation of 25 nm nanodisc. SEC profiles using Superpose 10/160 column. A) 4000:1 lipid :cNW50 shows predominantly liposomes/aggregation formation (EM photo showing Liposomes fraction, scale bar =200 nm). B) 2000:1 Lipid:cNW50 showed formation of predominantly 25 nm nanodisc. C) 1000:1 Lipid: cNW50 shows a peak for lipid free cNW50 (right) in addition to 25 nm nanodisc peak.

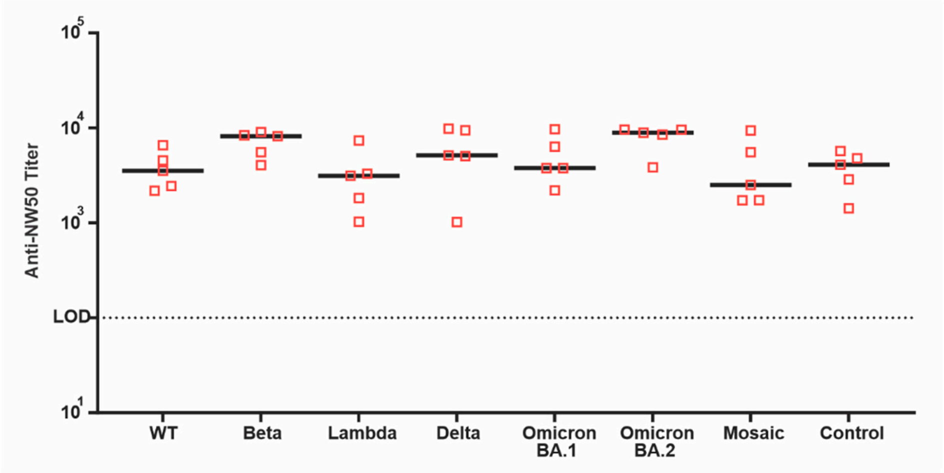

Figure S2. Anti NW50 IgG titer measured by ELISA. 1 μg mL^{-1} of NW50 protein diluted in coating buffer (28.5 mM Na2CO3; 71.4 mM NaHCO3, pH 9.6) was used to coat 96-well plates for 2 hr at 37 °C. Wells were washed and then blocked with 2% BSA in PBS containing 0.1% Tween-20 (PBS-T) for 2 hr at 37 °C. Mouse sera (serially diluted 10 folds in PBS-T containing 1% BSA) were incubated in the wells for 1 hr at 37 °C, then washed with PBS-T. Goat anti-mouse IgG-HRP was added. Wells were washed again with PBS-T before adding tetramethylbenzidine (TMB) solution. Antibody titers were defined as the reciprocal serum dilution at which the absorbance at 450 nm exceeded background by greater than 0.5 absorbance units.

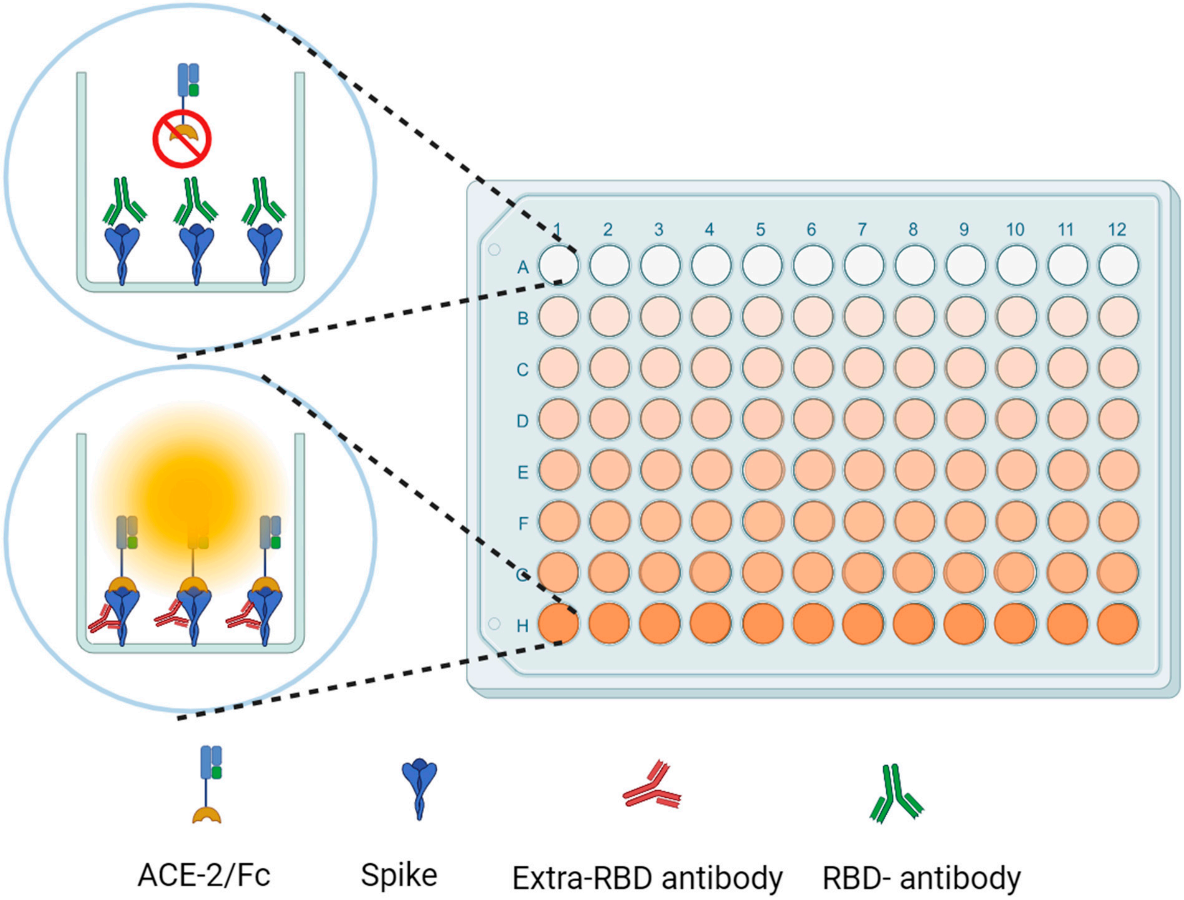

Figure S3. A schematic figure showing Spike/RBD-hACE2 Inhibition Assay: SARS-CoV-2 spike proteins was used to coat 96 well plates with 0.5µg/ml at 4 °C overnight and blocked with 2% BSA for 2 hours at 37 °C. The plate was used to check if post immune sera could block the interaction between hACE2-Fc and the coated spike protein.
